# Supplementary material for: Reactivation of associative structure specific outcome responses during prospective evaluation in reward-based choices
Source: Nat Commun. 2017 Jun 9;8:15821. doi: 10.1038/ncomms15821 (PMC5472730; doi:10.1038/ncomms15821)
Supplement: Supplementary Information [file ncomms15821-s1.pdf]

Type of file: pdf
Size of file: 0 KB
Title of file for HTML: Supplementary Information
Description: Supplementary Figures and Supplementary Note

Type of file: pdf
Size of file: 0 KB
Title of file for HTML: Peer Review File
Description:

SUPPLEMENTARY INFORMATION

Supplementary Figure 1:

a. Most rostral recording site:

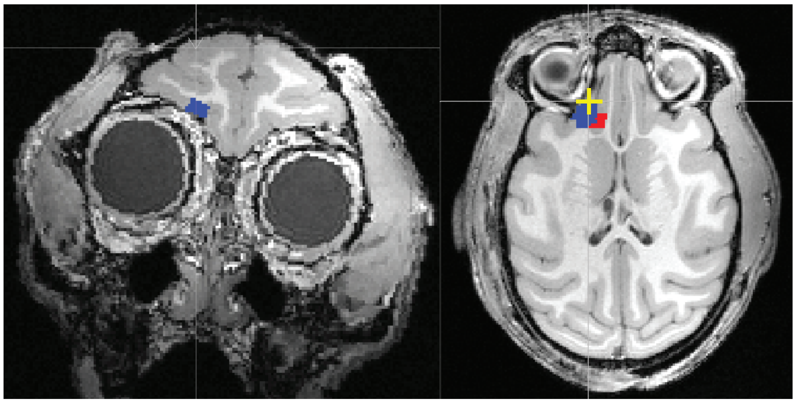

b. Most caudal recording site:

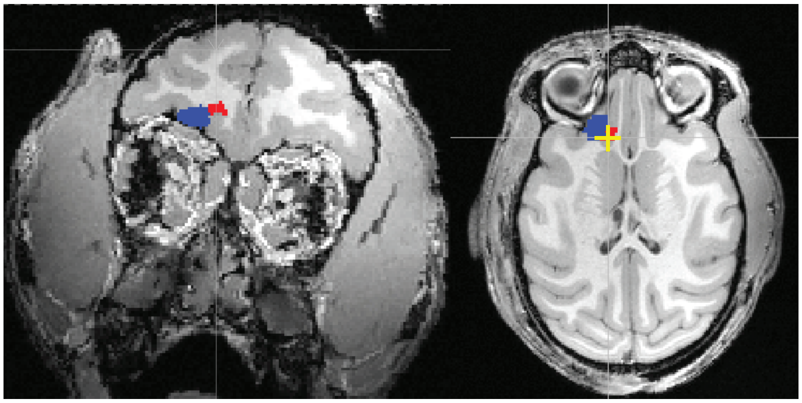

Supplementary Figure 1. Spread of Recording Site. All of our recordings are confined in Area 13 of OFC. The targeted area expands along the coronal planes situated between 28.65 mm and 33.60 mm rostral to the interaural plane with varying depth. Blue: Area 13m. Red: Area 13a. Yellow cross: most (a) rostral or (b) caudal recording site. On our recording grid (18-by-18 NAN grid), our data were collected from 16 out of 21 holes covering Area 13 of OFC with various depths for subject H and 15 out of 28 holes for subject B.

Supplementary Figure 2.

a. Modulation by Experienced Offer 1 for Cell # 69

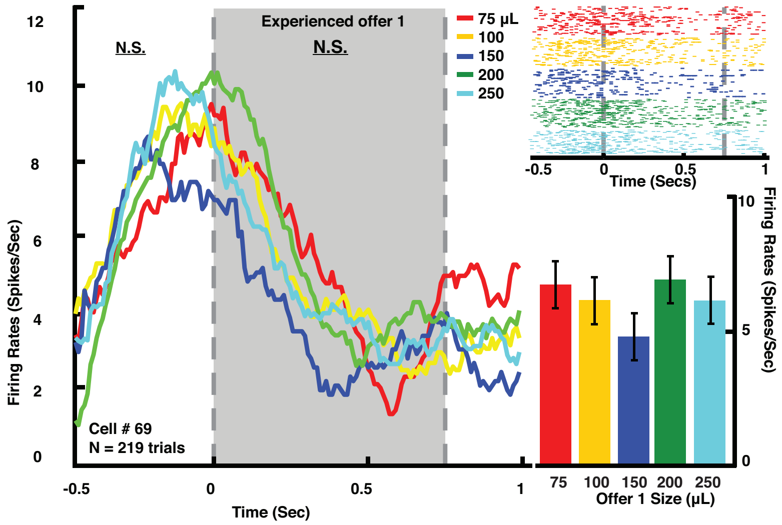

b. Modulation by Described Offer 1 for Cell # 123

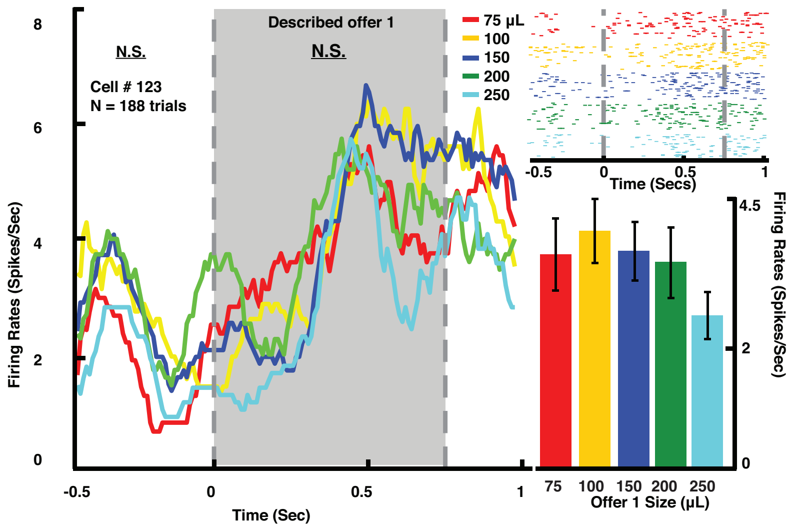

Supplementary Figure 2. Example Neurons. Left: Peristimulus time histogram (PSTH) for the example neuron. Top right: raster plot of the same example neuron; trials are sorted by offer 1 size. Bottom right: bar graph of the same example neuron showing averaged firing rates for each offer 1 size. **(a)** Activity of cell #69 is not significantly modulated by the size of experienced offer 1 even though this neuron is significantly modulated by the size of described offer 1, as shown in Figure 3a. **(b)** Activity of cell #123 is not significantly modulated by the size of described offer 1 even though this neuron is significantly modulated by the size of experienced offer 1, as shown in Figure 3b.

Supplementary Figure 3.

a. Cook's D between Regression Coefficients for Described Offer and Outcome

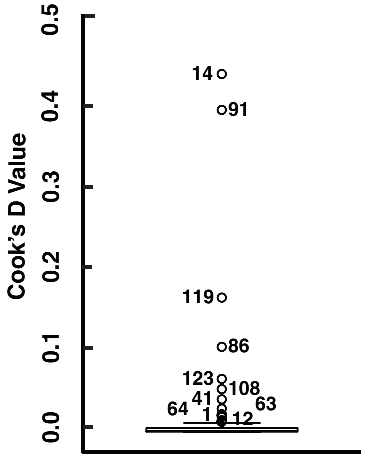

b. Cook's D between Regression Coefficients for Experienced Offer and Outcome

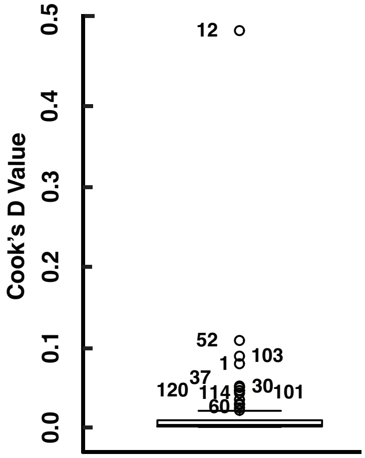

c. Cook's D between Regression Coefficients for Described and Experienced Offer

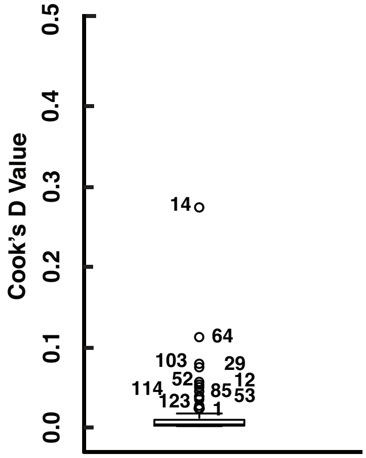

d. Cook's D between Regression Coefficients for Described and Experienced Outcome

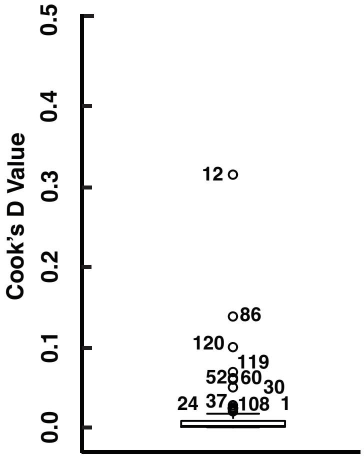

Supplementary Figure 3. Test for Outliers. Cook's D is used to measure the global influence (both discrepancy and leverage) for each pair of regression coefficient sets in the correlation analyses for the key hypothesis testing in Figure 4. Each dot and the number immediately to it represent a neuron with Cook's D value larger than the third quartile of the entire sample. No data point has a value of Cook's D ≥ 1, which means no data point in our sample qualifies as outlier.

Supplementary Figure 4.

a. Encoding of Described Offer 1 and Outcome

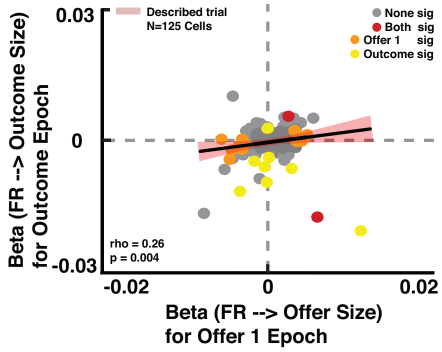

b. Permutation Test for Coding Format Correlation between Described Offer 1 and Outcome

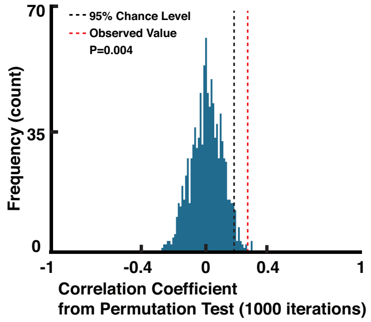

c. Encoding of Experienced Offer 1 and Outcome

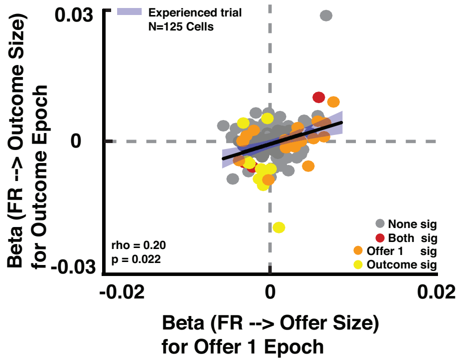

d. Permutation Test for Coding Format Correlation between Experienced Offer 1 and Outcome

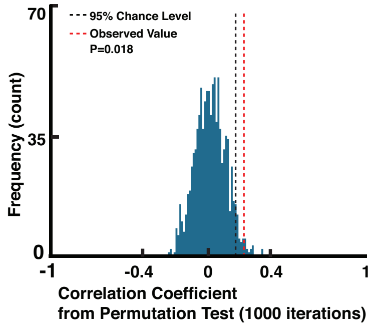

Supplementary Figure 4. Reactivation Response Measured with Alternative Regression Models. The alternative regression model for outcome epoch includes all trials and a controlling factor of choice. **(a, c)** Each dot represents a neuron. Orange: the neuron is significantly tuned in the regression described on the x-axis. Yellow: the neuron is significantly tuned in the regression described on the y-axis. Red: the neuron is significantly tuned in the regressions described on both the x-axis and the y-axis. Gray: the neuron is not significantly tuned in either the regression described on the x-axis or the y-axis. **(a)** Left: neural encoding for described offers closely resembled that for described-trial outcomes, after controlling for encoding for choice during outcome epoch. Right: permutation test results. **(b)** Left: neural encoding for experienced offers closely resembled that for experienced-trial outcomes, even after controlling for encoding for choice during outcome epoch. Right: permutation test results.

Supplementary Figure 5.

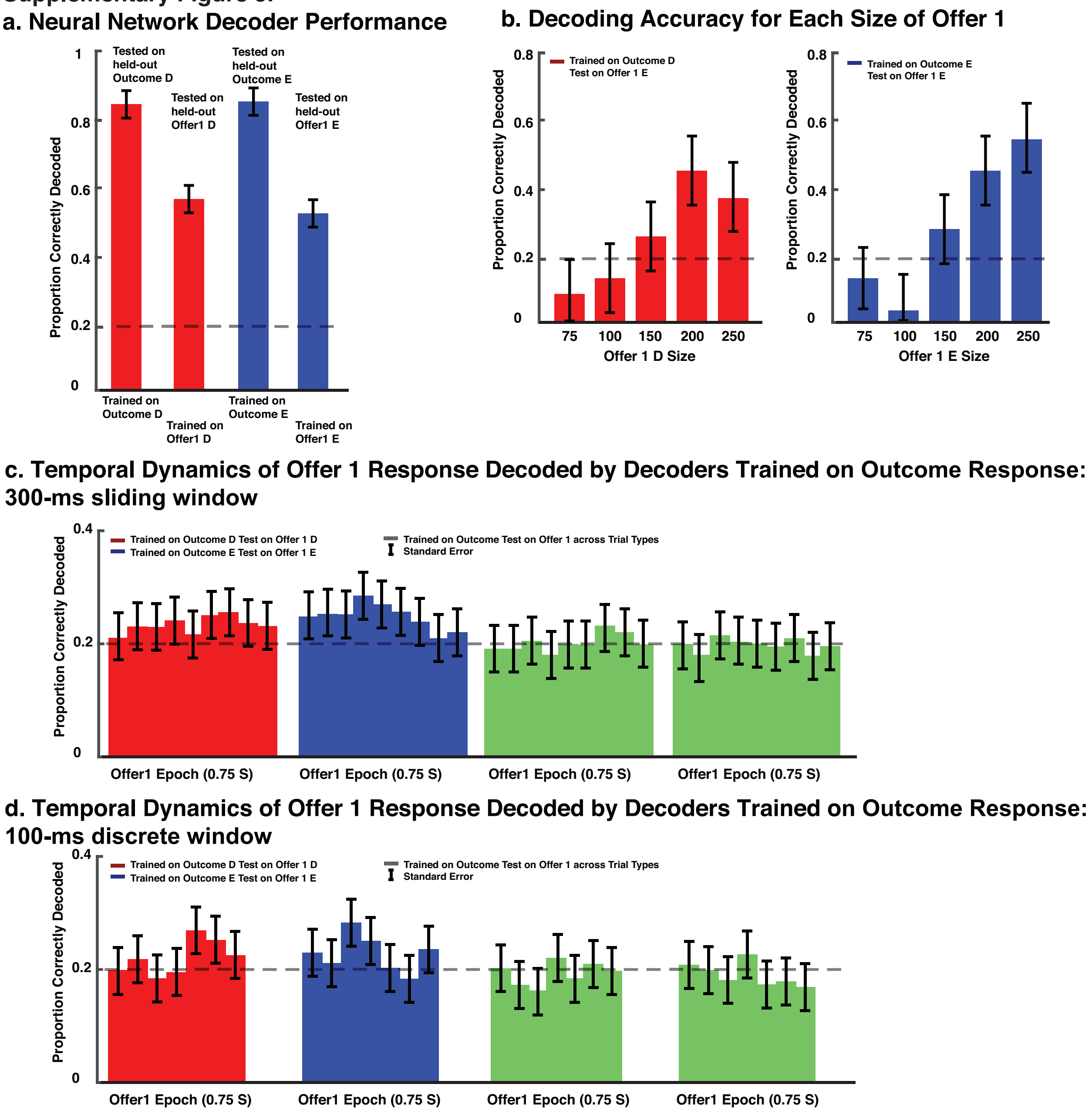

Supplementary Figure 5. Decoding Results of the Neural Network Decoders. D: described trials. E: experienced trials. **(a)** A held-out set of population activation patterns was used to test the performance of the decoder. In all cases, the decoders can successfully decode the held-out test sets of population activation patterns from the same epoch (offer 1 vs. outcome) and context (described vs. experienced) as the training sets. This decoder performance can also be used as an approximation of the highest performance achievable when testing a decoder with neural response from an epoch it was never trained on (as shown in Figure 5a). **(b)** The relatively low, although significant, decoding accuracy, was due to low decoding accuracy on smaller-sized offers for which a small set of neurons had no corresponding data in outcome epoch, since the monkeys seldom chose and received the smaller-sized offers. **(c-d)** Temporal dynamics of offer 1 response decoded by decoders trained on outcome response. Coding of described offers closely resembled that of outcome during a slightly later time point of offer 1 presentation. Coding of experienced offers closely resembled that of outcome during a slightly earlier time point of offer 1 presentation. **(c)** Population activation patterns of offer 1 were organized into 300-ms sliding windows throughout the entire time of offer 1 presentation. **(d)** Population activation patterns of offer 1 were organized into 100-ms discrete windows throughout the entire time of offer 1 presentation.

SUPPLEMENTARY NOTE 1

We observed asymmetry in cross-decoding between neural activation states to outcomes in described and in experienced trials. This asymmetry is likely due to a high level of noise in training data (i.e. neural activation to experienced outcomes). The fact that subjects made free choices means that they seldom chose and received smaller sized offers (they, not surprisingly, preferred the larger offer). Therefore, some neurons have few trials corresponding to choosing and receiving smaller-sized reward as outcome. It is more so in experienced trials, where subjects were more reward maximizing than in described trials (Figure 2 in the main text).
